# Supplementary material for: Human and Animal Brucellosis in Nigeria: A Systemic Review and Meta-Analysis in the Last Twenty-One Years (2001–2021)
Source: Vet Sci. 2022 Jul 26;9(8):384. doi: 10.3390/vetsci9080384 (PMC9394481; doi:10.3390/vetsci9080384)
Supplement: Supplementary file 1 [file vetsci-09-00384-s001.zip › vetsci-1803405-supplementary.pdf]

Supplementary Table S1. Published report on Human and Animal brucellosis in Nigeria (2001–2021).

Zone (Region)	State	Reference	Years of study	Sample source	Type of sample	No of sample	Positive sample	Method of detection	Specie type				
									Brucella spp	B. abortus	B. melitensis	B. suis	B. canis
NC	FCT	[14]	2017	Cattle	blood	376	21	RBPT, cELISA,	21	0	0	0	0
				Goat	blood	203	19	RBPT, cELISA,	19	0	0	0	0
				Sheep	blood	260	113	RBPT, cELISA,	113	0	0	0	0
	Benue	[15]	2011 and 2013	Goat	blood	331	57	RBPT, Culture,	0	57	0	0	0
	Plateau	[16]	2010	Horse	blood	22	5	RBPT	0	5	0	0	0
	Niger	[17]	2016	Cattle	blood	672	13	LFA	0	13	0	0	0
	Plateau	[18]	2009	Sheep	blood	496	72	RBPT, SAT	72	0	0	0	0
				Goat	blood	851	137	RBPT, SAT	137	0	0	0	0
	Kwara	[19]	2020	Human	blood	189	42	RBPT, iELISA,	42	0	0	0	0
				Cattle	blood	394	38	RBPT, iELISA,	38	0	0	0	0
	Kwara	[20]	2019	Horse	blood	48	8	RBPT	8	0	0	0	0
	Plateau	[21]	2020	Cattle	vaginal swab	70	11	Culture	0	11	0	0	0
					milk	50	0	Culture	0	0	0	0	0
					hygroma fluid	2	0	Culture	0	0	0	0	0
	Benue	[22]	2018	Dog	blood	102	2	RBT, SAT	2	0	0	0	0
	Plateau	[23]	2020	Cattle	milk	200	27	MSA	27	0	0	0	0
	Plateau	[24]	2011	Cattle	blood	158	6	RBPT	0	6	0	0	0
	Plateau	[25]	2014	Dog	blood	350	113	RBPT	113	0	0	0	0
	Plateau	[26]	2017	Cattle	blood	479	18	RBPT, SAT	18	0	0	0	0
	Plateau	[27]	2014	Sheep	blood	50	14	RBPT	14	0	0	0	0
				Goat		50	31	RBPT	31	0	0	0	0
	Niger	[28]	2011	Cattle	Blood	87	9	RBPT	0	9	0	0	0
	Nasarawa	[29]	2017	Goat/sheep	blood	256	64	RBPT, cELISA,	64	0	0	0	0
	FCT	[30]		Human	Blood	224	40	RBPT, IgG, IgM ELISA	40	0	0	0	0
	Nasarawa	[31]	2018	Human	Blood	160	16	RBPT	16	0	0	0	0
	Benue	[32]	2013	Pig	Blood	281	86	RBPT	86	0	0	0	0
	Plateau	[33]	2015	Horse	blood	7	7	RBPT, SAT	0	7	0	0	0
				Cattle	blood	44	19	RBPT, SAT	0	19	0	0	0
				Sheep	blood	32	4	RBPT, SAT	0	4	0	0	0
				Goat	blood	50	5	RBPT, SAT	0	5	0	0	0
				Cattle	milk	12	3	Culture	0	3	0	0	0
				Sheep	milk	10	0	Culture	0	0	0	0	0
				Goat	milk	10	0	Culture	0	0	0	0	0
				Horse	vaginal swab	6	2	Culture	0	2	0	0	0

				Cattle	vaginal swab	12	0	Culture	0	0	0	0	0
				Sheep	vaginal swab	10	0	Culture	0	0	0	0	0
				Goat	vaginal swab	10	0	Culture	0	0	0	0	0
	Benue	[34]	2015	Cattle	milk	102	16	MRT	16	0	0	0	0
	FCT	[35]	2010	Avian spp	blood	275	10	RBPT	10	0	0	0	0
				Sheep	blood	203	19	RBPT, SAT	19	0	0	0	0
				Goat	blood	260	51	RBPT, SAT	51	0	0	0	0
	Plateau	[36]		Cattle	Aborted fetuses	10	1	Culture	0	1	0	0	0
				Cattle	Vaginal swabs	129	0	Culture	0	0	0	0	0
				Cattle	milk	60	5	Culture	0	5	0	0	0
				Cattle	blood	17	0	Culture	0	0	0	0	0
				Cattle	hygroma fluid	1	0	Culture	0	0	0	0	0
				Sheep	Aborted fetuses	2	0	Culture	0	0	0	0	0
				Sheep	Vaginal swabs	34	0	Culture	0	0	0	0	0
				Sheep	milk	10	0	Culture	0	0	0	0	0
				Goat	milk	8	0	Culture	0	0	0	0	0
				Goat	Vaginal swabs	7	0	Culture	0	0	0	0	0
				Dog	Aborted fetuses	5	0	Culture	0	0	0	0	0
				Dog	blood	31	0	Culture	0	0	0	0	0
				Dog	milk	4	0	Culture	0	0	0	0	0
				Horse	Vaginal swabs	2	1	Culture	0	1	0	0	0
				Horse	hygroma fluid	1	1	Culture	0	1	0	0	0
				Pig	Aborted fetuses	3	0	Culture	0	0	0	0	0
	Nasarawa	[36]		Cattle	Vaginal swabs	7	0	Culture	0	0	0	0	0
				Cattle	milk	2	1	Culture	0	1	0	0	0
				Cattle	hygroma fluid	1	1	Culture	0	1	0	0	0
				Cattle	blood	0	0	Culture	0	0	0	0	0
	Kogi	[36]		Cattle	Vaginal swabs	156	0	Culture	0	0	0	0	0
				Cattle	milk	2	0	Culture	0	0	0	0	0
				Cattle	hygroma fluid	0	0	Culture	0	0	0	0	0
				Cattle	blood	0	0	Culture	0	0	0	0	0
	Plateau	[37]	2019	cattle	blood	170	88	iELISA, LFA, Culture	0	59	29	0	0
				Sheep	blood	201	100	iELISA, LFA, Culture	0	0	100	0	0
				Goat	blood	98	46	iELISA, LFA, Culture	0	0	46	0	0
				Swine	blood	85	39	iELISA, LFA, Culture	0	0	0	39	0
Total						**8450**	**1381**		**957**	**210**	**175**	**39**	**0**

NE	Borno	[38]	2013-2014	Donkey	blood	601	43	RBPT, iELISA,	43	0	0	0	0
	Yobe	[39]	2020	Donkey	blood	200	43	RBPT, cELISA,	43	0	0	0	0
	Adamawa	[40]	2008-2009	Cattle	blood	87	192	RBPT	192	0	0	0	0
	Borno	[41]	2014	CHICKEN	blood	556	10	RBPT, MAT	10	0	0	0	0
				G. fowl	blood	84	6	RBPT, MAT	6	0	0	0	0
				DUCK	blood	50	2	RBPT, MAT	2	0	0	0	0
				TURKEY	blood	40	0	RBPT, MAT	0	0	0	0	0
	Bauchi	[42]	2001	sheep	blood	28	4	RBPT, SAT	4	0	0	0	0
					milk	5	5	MRT, Culture	5	0	0	0	0
				Ewe	vaginal swab	5	3	Culture	3	0	0	0	0
				Cattle	blood	20	20	RBPT	20	0	0	0	0
	Borno	[15]	2011 and 2013	Goat	blood	195	4	RBPT	4	0	0	0	0
	Taraba	[43]	2018	Goat	blood	386	37	RBPR	37	0	0	0	0
	Gombe	[44]	2016	Cattle	blood	200	18	RBPT, SAT, MAT	0	18	0	0	0
	Not reported	[45]	2001	Human	blood	500	26	SAT	26	0	0	0	0
	Taraba	[46]		Horse	blood	100	16	RBPT, SAT	16	0	0	0	0
	Borno	[47]	2010	Carmel	blood	511	62	RBPT, MSA	62	0	0	0	0
	Adamawa	[48]	2008-2009	Cattle	blood	1827	332	RBPT, cELISA,	332	0	0	0	0
	Bauchi	[49]	2019	Cattle	blood	1000	235	RBPT, cELISA,	235	0	0	0	0
	Borno	[50]	2017	Human	blood	106	4	RBPT	4	0	0	0	0
	Borno	[51]	2010	Camel	blood	257	38	RBPT, SAT	38	0	0	0	0
	Gombe	[52]	2017	Dog	blood	374	76	RBPT, cELISA,	0	76	0	0	0
	Bauchi	[53]	2013-2014	Cattle	blood	366	18	RBPT, SAT	18	0	0	0	0
	Yobe	[54]		Camel	blood	400	298	MSAT	298	0	0	0	0
	Yobe	[55]	2013	Donkey	blood	300	15	RBPT, cELISA, MSAT	15	0	0	0	0
	Borno	[56]	2015	Human	Blood	100	11	RBPT	0	11	0	0	0
	Bauchi	[57]	2020	Human	Blood	284	95	RBPT, IgG ELISA, IgM ELISA, IgG/IgM	95	0	0	0	0
	Borno	[58]	2016	Carmel	blood	99	0	RBPT, LFA	0	0	0	0	0
	Borno	[59]	2012	Goat	blood	250	34	RBPT, CFT	0	12	22	0	0
	Sokoto	[60]	2008	Cattle	blood	1547	305	RBPT, cELISA, SAT	305	0	0	0	0
					milk	99	25	MRT	25	0	0	0	0
	Bauchi	[36]		Cattle	Vaginal swabs	5	3	Culture	0	3	0	0	0
				Cattle	milk	5	2	Culture	0	2	0	0	0
				Cattle	hygroma fluid	0	0	Culture	0	0	0	0	0
				Cattle	blood	0	0	Culture	0	0	0	0	0
				Sheep	Vaginal swabs	0	0	Culture	0	0	0	0	0
				Sheep	milk	3	2	Culture	0	2	0	0	0
	Adamawa	[36]		Cattle	Vaginal swabs	3	0	Culture	0	0	0	0	0
				Cattle	milk	3	0	Culture	0	0	0	0	0
				Cattle	hygroma fluid	3	2	Culture	0	2	0	0	0

				Cattle	blood	0	0	Culture	0	0	0	0	0
				Sheep	vaginal swabs	9	0	Culture	0	0	0	0	0
	Taraba	[36]		Cattle	Vaginal swabs	0	0	Culture	0	0	0	0	0
				Cattle	milk	0	0	Culture	0	0	0	0	0
				Cattle	hygroma fluid	3	2	Culture	0	2	0	0	0
				Cattle	blood	0	0	Culture	0	0	0	0	0
	Borno	[36]		Cattle	Vaginal swabs	48	0	Culture	0	0	0	0	0
				Cattle	milk	37	0	Culture	0	0	0	0	0
				Cattle	hygroma fluid	0	0	Culture	0	0	0	0	0
				Cattle	blood	0	0	Culture	0	0	0	0	0
				Sheep	Vaginal swabs	7	0	Culture	0	0	0	0	0
	Bauchi	[61]	2019	Donkey	blood	1000	114	RBPT, SAT	114	0	0	0	0
		[62]	2011-2013	Cattle	blood	*	*						
Total						**11,703**	**2102**		**1952**	**128**	**22**	**0**	**0**
NW	Katsina	[63]	2017	Camel	blood	980	110	RBPT, SAT	110	0	0	0	0
	kaduna	[64]	2018	Cattle	blood	400	74	RBPT, cELISA,	74	0	0	0	0
	Kaduna	[40]	2008-2009	Cattle	blood	98	0	RBPT	0	0	0	0	0
	Kano	[40]	2008-2009	Cattle	blood	66	0	RBPT	0	0	0	0	0
	Sokoto	[15]	2011 and 2013	Goat	blood	146	0	RBPT	0	0	0	0	0
	Kaduna	[16]	2010	Horse	blood	53	6	RBPT	0	6	0	0	0
	Sokoto	[62]	2011-2013	Cattle	blood	2356	90	RBPT	0	90	0	0	0
	kaduna	[65]		Goat	blood	422	114	RBPT, SAT, LFA	114	0	0	0	0
	kaduna	[66]	2007	Human	blood	1	1	cELISA	0	1	0	0	0
	Jigawa	[67]		Cattle	blood	570	23	RBPT, cELISA,	23	0	0	0	0
	Sokoto	[68]		Sheep	blood	720	170	RBPT, SAT, cELISA	170	0	0	0	0
	kaduna	[69]	2016	Cattle	blood	500	14	RBPT, SAT	14	0	0	0	0
	kaduna	[48]	2008-2009	Cattle	blood	1048	892	RBPT, cELISA,	892	0	0	0	0
	KANO	[48]	2008-2009	Cattle	blood	1870	511	RBPT, cELISA,	511	0	0	0	0
	kaduna	[70]	2012	Sheep	blood	5	1	RBPT, SAT	0	1	0	0	0
	Kaduna	[21]	2020	Cattle	vaginal swab	63	0		0	0	0	0	0
					milk	36	0		0	0	0	0	0
					hygroma fluid	2	0		0	0	0	0	0
	Sokoto	[71]		Horse	Blood	347	48	RBPT, SAT, cELISA	48	0	0	0	0
	kaduna	[72]	2017	Human	blood	100	19	iELISA,	19	0	0	0	0
	kaduna	[73]	2018	Sheep	blood	400	100	cELISA	0	57	43	0	0
	Jigawa	[74]	2020	Cattle	blood	1810	61	SAT	61	0	0	0	0
	kaduna	[75]		Sheep	Blood	17	13	RBPT, SAT	13	0	0	0	0
	Sokoto	[76]	2021	Human	Blood	137	1	RBPT, cELISA,	0	1	0	0	0
				Cattle	Blood	366	19	RBPT, cELISA,	0	19	0	0	0
	Sokoto	[77]	2019	Sheep	blood	169	40	cELISA	0	0	0	40	0

	Katsina	[77]	2019	Sheep	blood								
	Sokoto	[77]	2019	Goat	blood	231	28	cELISA	0	0	0	28	0
	Katsina	[77]	2019	Goat	blood								
	Sokoto	[78]	2019	Cattle	lymph node	170	50	PCR	0	50	0	0	0
	Sokoto	[79]	2006	Cattle	blood	1711	395	RBPT, SAT, cELISA,	395	0	0	0	0
	Kaduna	[80]	2017	Goat	blood	768	39	RBPT, SAT	39	0	0	0	0
				Sheep	blood	268	27	RBPT, SAT	27	0	0	0	0
	Kaduna	[81]	2014-2015	Goat	blood	280	23	RBPT, cELISA,	23	0	0	0	0
					milk	113	43	MRT	43	0	0	0	0
	Taraba	[82]	2014	cattle	blood	150	32	RBPT	0	32	0	0	0
				Goat	blood	90	10	RBPT	0	10	0	0	0
				Sheep	blood	90	18	RBPT	0	18	0	0	0
				Cow	milk	225	17	MRT	0	17	0	0	0
	Jigawa	[83]	2017	Cattle	blood	1867	83	SAT	83	0	0	0	0
					milk	869	61	WAT	61	0	0	0	0
	Zamfara	[84]		Cattle	blood	320	64	iELISA,	0	64	0	0	0
	Kaduna	[85]	2015	Sheep	blood	579	153	RBPT, SAT, LFA	153	0	0	0	0
	kaduna	[86]	2003	sheep	milk	94	14	MRT	14	0	0	0	0
				Goat	milk	60	24	MRT	24	0	0	0	0
	Katsina	[86]		Sheep	milk	48	6	MRT	6	0	0	0	0
				Goat	milk	10	1	MRT	1	0	0	0	0
	Kano	[86]		Sheep	milk	59	20	MRT	20	0	0	0	0
				Goat	milk	12	4	MRT	4	0	0	0	0
	Sokoto	[86]		Goat	milk	59	17	MRT	17	0	0	0	0
	Kano	[58]	2016	Camel	blood	180	4	RBPT, LFA	0	4	0	0	0
	Sokoto	[86]				32	2	RBPT, LFA	0	2	0	0	0
	Kaduna	[87]		Cattle	blood	2799	198	RBPT, SAT	198	0	0	0	0
					milk	2149	31	MRT	31	0	0	0	0
	Kaduna	[88]	2004	Dog	blood	200	43	RBPT, SAT	0	43	0	0	0
	Kaduna	[36]	2004	Cattle	Vaginal swabs	3	0	Culture	0	0	0	0	0
				Cattle	milk	5	0	Culture	0	0	0	0	0
				Cattle	hygroma fluid	0	0	Culture	0	0	0	0	0
				Cattle	blood	0	0	Culture	0	0	0	0	0
	Kano	[36]	2004	Cattle	Vaginal swabs	23	0	Culture	0	0	0	0	0
				Cattle	milk	17	0	Culture	0	0	0	0	0
				Cattle	hygroma fluid	0	0	Culture	0	0	0	0	0
				Cattle	blood	60	0	Culture	0	0	0	0	0
	Sokoto	[36]	2004	Cattle	Vaginal swabs	72	0	Culture	0	0	0	0	0
				Cattle	milk	6	2	Culture	0	2	0	0	0
				Cattle	hygroma fluid	0	0	Culture	0	0	0	0	0

				Cattle	blood	17	0	Culture	0	0	0	0	0
Total						**26,318**	**3716**		**3188**	**417**	**43**	**68**	**0**
SE	Enugu	[89]	2018	Cattle	blood	484	14	RBPT	14	0	0	0	0
				Goat	blood	340	12	RBPT	12	0	0	0	0
	Ebonyi	[62]	2011-2013	Cattle	blood	1082	37	RBPT	0	37	0	0	0
	Enugu	[90]	2020	Dog	blood	68	34	Immunocomb	0	0	0	0	34
	Anambra	[90]	2020	Dog	blood	55	0	Immunocomb	0	0	0	0	0
	Enugu	[91]	2015-2016	Human	Blood	682	195	RBPT	195	0	0	0	0
	Anambra	[92]		Chicken	blood	410	14	RBPT	14	0	0	0	0
	Anambra	[93]	2011	pig	blood	139	0	RBPT	0	0	0	0	0
	Enugu	[93]	2011	pig	blood	183	2	RBPT	2	0	0	0	0
	Ebonyi	[93]	2011	pig	blood	29	0	RBPT	0	0	0	0	0
	Enugu	[36]		Cattle	hygroma fluid	1	0	Culture	0	0	0	0	0
	Enugu	[94]	2018	horse	blood	402	12	RBPT	12	0	0	0	0
Total						**3875**	**320**		**249**	**37**	**0**	**0**	**34**
SS	Edo	[95]		Pig	blood	30	81	Culture	0	24	27	30	0
					swab	25	68	Culture	0	19	24	25	0
	Akwa Ibom	[96]	2019	Human	Blood	228	70	RBPT, IgG-IgM-ELISA	0	0	70	0	0
	C. River	[97]	2013	Cattle	blood	354	149	RBPT, Culture	149	0	0	0	0
					Vaginal swabs	14	1	Culture	0	1	0	0	0
					hygroma fluid	1	1	Culture	0	1	0	0	0
	Edo	[62]	2011-2013	Cattle	blood	*	*						
Total						**652**	**370**		**149**	**45**	**121**	**55**	**0**
SW	Lagos	[98]	2011	DOG	blood	183	18	RBPT, RSA	0	17	0	0	1
	Oyo	[98]	2011	DOG	blood	180	3	RBPT, RSA	0	3	0	0	0
	Oyo	[99]	2013	Cattle	blood	1,241	97	RBPT, iELISA,	97	0	0	0	0
					milk	57	19	RBPT, iELISA, MRT	19	0	0	0	0
	Oyo	[15]	2011 and 2013	Goat	blood	2155	13	RBPT	13	0	0	0	0
	Lagos	[62]	2011-2013	Cattle	blood	4667	188	RBPT	0	188	0	0	0
	Ogun					*							
	Oyo					*							
	Lagos	[100]	2018	Cattle	blood	221	38	RBPT, iELISA,	38	0	0	0	0
				Goat	blood	192	29	RBPT, iELISA,	29	0	0	0	0
				sheep	blood	60	14	RBPT, iELISA,	14	0	0	0	0
	Oyo	[7]	2018	Cattle	blood	149	17	RBPT, cELISA,	17	0	0	0	0

	ogun	[101]		Cattle	blood	279	24	RBPT, cELISA,	24	0	0	0	0
	Oyo	[102]		Cattle	blood	174	6	RBPT, cELISA,	0	6	0	0	0
	Oyo	[103]	2008	Cattle	cow milk	532	99	RBPT, MRT	0	99	0	0	0
					blood	532	52	RBPT	0	52	0	0	0
	Oyo	[104]	2004-2006	Cattle	blood	1642	97	RBPT	97	0	0	0	0
	Lagos	[105]	2017	Human	blood	422	27	RBPT, iELISA,	27	0	0	0	0
	Lagos	[106]	2009	Cattle	blood	479	41	RBPT	41	0	0	0	0
	Oyo	[107]	2015	Cattle	blood	220	12	RBPT	12	0	0	0	0
	Sw	[108]	2018	Cattle	blood	513	52	RBPT, cELISA,	0	52	0	0	0
					milk	635	130	RBPT, iELISA,	0	130	0	0	0
	Oyo	[35]	2010	Avian spp	blood	140	5	RBPT	5	0	0	0	0
	Ogun	[109]	2011-2014	Dogs	blood	354	71	RBPT, RSA	71	0	0	0	0
	Lagos	[109]	2011-2014	Dogs	blood	385	84	RBPT, RSA	84	0	0	0	0
	oyo	[110]	2004	Cattle	blood	1117	65	RBPT	65	0	0	0	0
				Sheep	blood	34	0	RBPT	0	0	0	0	0
				Goat	blood	466	4	RBPT	4	0	0	0	0
				Pig	blood	200	0	RBPT	0	0	0	0	0
				Human	blood	11	7	RBPT	7	0	0	0	0
Total						**17240**	**1212**		**664**	**547**	**0**	**0**	**1**
Grand Total						**68,238**	**9101**		**7159**	**1384**	**361**	**162**	**35**

RBPT: Rose Bengal Plate Test, **SAT**: Serum Agglutination Test, **cELISA**: competitive enzyme linked immunosorbent assay, **iELISA**: Indirect enzyme linked immunosorbent assay, **MRT**: Milk Ring Test, **CFT**: complement fixation test**, MSAT:** Microtiter Serum Agglutination Test, **IgM**: immunoglobulin M, **IgG**: immunoglobulin G, **IBCA:** Immunocomb WAT: Wright agglutination test **LFA**: Lateral flow assay **RSA**: Rapid slide test, **MAT**: Micro-agglutination test
